# Activating the Basal Planes and Oxidized Oxygens in Layer‐Structured Na_0.6_CoO_2_ for Boosted OER Activity

**DOI:** 10.1002/advs.202305959

**Published:** 2023-11-30

**Authors:** Bing Xiong, Tingxin Fu, Qiuping Huang, Jianlin Wang, Zhangzhang Cui, Zhengping Fu, Yalin Lu

**Affiliations:** ^1^ CAS Key Laboratory of Materials for Energy Conversion Department of Materials Science and Engineering University of Science and Technology of China Hefei Anhui 230026 China; ^2^ Anhui Laboratory of Advanced Photon Science and Technology University of Science and Technology of China Hefei Anhui 230026 China; ^3^ Synergetic Innovation Center of Quantum Information & Quantum Physics Hefei National Research Center for Physical Sciences at the Microscale University of Science and Technology of China Hefei Anhui 230026 China

**Keywords:** active areas, layered oxides, oxygen evolution reaction, oxidized oxygens

## Abstract

With the CoO_2_ slabs consisting of Co_4_O_4_ cubane structure, layered Na*_x_* CoO_2_ are considered promising candidates for oxygen evolution reaction (OER) in alkaline media given their earth‐abundant and structural advantages. However, due to the strong adsorption of intermediates on the large basal planes, Na*_x_* CoO_2_ cannot meet the activity demands. Here, a novel one‐pot synthesis strategy is proposed to realize the high solubility of iron in Na*_x_* CoO_2_ in an air atmosphere. The optimist Na_0.6_Co_0.9_Fe_0.1_O_2_ exhibits enhanced OER activity compared to their pristine and other reported Fe‐doped Na*_x_* CoO_2_ counterparts. Such an enhancement is mainly ascribed to the abundant active sites on the activated basal planes and the participation of oxidized oxygen as active sites independently, which breaks the scaling relationship limit in the OER process. This work is expected to contribute to the understanding of the modification mechanism of Fe‐doped cobalt‐based oxides and the exploitation of layer‐structured oxides for energy application.

## Graphical abstract

The incorporation of Fe into CoO_2_ slabs significantly improve the oxygen evolution reaction (OER) activity of layered Na_0.6_CoO_2_. The enhancement is mainly ascribed to the enriched active sites on the activated basal planes and the participation of oxidized oxygen as active sites independently, which breaks the scaling relationship limit in the OER process.

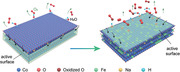

## 1 Introduction

Electrochemical water electrolyzers are promising energy storage/conversion systems to utilize intermittent electricity from renewable energies, such as solar, wind, biomass, and hydropower.^[^ ^1^, ^2^ ^]^ Recently, the reduction in the cost of renewable energy generation and the possibility of coupling electrolyzers directly with clean energy sources have made water electrolysis more economically and technically appealing.^[3]^ The anodic oxygen evolution reaction (OER) remains the bottleneck of the water splitting due to the sluggish four‐electron step, which limits the overall efficiency of the electrolysis and hampers broader industrial applications. Although ruthenium and iridium oxides achieve favorable catalytic performance, the prohibitive cost and poor stability have hindered their use as large‐scale commercial catalysts. Therefore, the development of efficient and economical OER electrocatalysts is an urgent priority for both fundamental research and industrial applications under the context of carbon neutrality and hydrogen economy.

Up to now, many types of noble metal‐free oxides, such as perovskites,^[^ ^4^, ^5^, ^6^, ^7^ ^]^ spinels,^[^ ^8^, ^9^, ^10^ ^]^ hydroxides,^[^ ^11^, ^12^, ^13^ ^]^ and other oxides^[^ ^14^, ^15^, ^16^ ^]^ have been studied and have achieved excellent OER performance. Among 3d transition metal oxides, cobalt‐based materials were found to be highly attractive OER electrocatalysts due to their highly tunable physicochemical properties and catalytic performance. And octahedral Co (Co_Oh_) has long been identified as critical active sites for OER in Co‐based perovskite and spine oxides.^[^ ^17^, ^18^, ^19^ ^]^ Recently, researchers highlighted that the high OER activity is strongly correlated to di‐μ‐oxo bridged Co_4_O_4_ cubane structure, in which the direct Co_Oh_ couplings can facilitate the formation of O─O bonds.^[^ ^20^, ^21^ ^]^ Meanwhile, we demonstrated that the surface termination of (111)‐facet Co_3_O_4_ with exclusive oxo‐bridged Co_Oh_ is much more active than its non‐faceted counterpart.^[22]^ Layer‐structured A*_x_* CoO_2_ (A = Li, Na, K) oxides are alternately stacked by the layers of CoO_2_ and alkali ions, with the CoO_2_ layers consisting of edge‐sharing CoO_6_ octahedra,^[23]^ the di‐μ‐oxo bridged Co_Oh_ motifs in A_x_CoO_2_ imply a potential structural advantage over perovskites and spinels.

On the other hand, Lu et al. demonstrated that the edge sites of the CoO_2_ slabs in Na_x_CoO_2_ are highly active for OER, while the adsorption of reaction intermediates on the basal planes is too strong.^[24]^ The large inert basal plane endows Na_x_CoO_2_ with unsatisfactory OER performance. Recently, it has been reported that the introduction of Fe into Na_x_CoO_2_ (direct synthesis or dynamic incorporation) significantly enhances its OER catalytic activity.^[^ ^25^, ^26^, ^27^ ^]^ Using experimental characterizations and DFT calculations, researchers ascribed the increased activity to a synergistic effect, dual‐metal‐site mechanism, and charge redistribution. Notwithstanding the above advances, the identification of active areas and the role of oxygen species are often overlooked. On the other hand, the high‐Fe doping content can only be realized in the protective atmosphere or with the introduction of magazine metal elements, while only 5% substitution of Fe can be achieved in Na_x_CoO_2_ synthesized in air atmosphere.^[^ ^26^, ^27^ ^]^ The extremely limited solubility hinders the exploration of optimal doping content and deeper mechanism analysis. Accordingly, the development of a synthetic method for mass production of high Fe‐doping content and the study of the nature of enhanced activity are important tasks in the development of layered Na_x_CoO_2_ into practical electrocatalysts.

Here, using NaOH as the Na source, we successfully synthesized Na_0.6_Co_1‐x_Fe_x_O_2_ (denoted as NCFx, x = 0≈0.25) with high Fe content. ECSA, LSV, and kinetic studies have shown that Fe activates the Co sites, but does not act as the active sites in Na_0.6_Co_1‐x_Fe_x_O_2_. Furthermore, we speculate that Fe can optimize the adsorption of intermediates on basal planes according to DOS calculations. The subsequent acid‐etching, morphology analysis, O_2_‐TPD profiles, and electrochemical measurements corroborate our hypothesis. That is, the introduction of Fe initiates the transition of the active sites from edge areas to large basal planes. Moreover, spectroscopic analysis, charge calculations, and pH‐dependent experiments suggest that the oxidized oxygen can be activated to serve as active sites independently and break the scaling relationship limit (SRL) in most OER processes.

## 2 Results and Discussion

**Figure 1:**
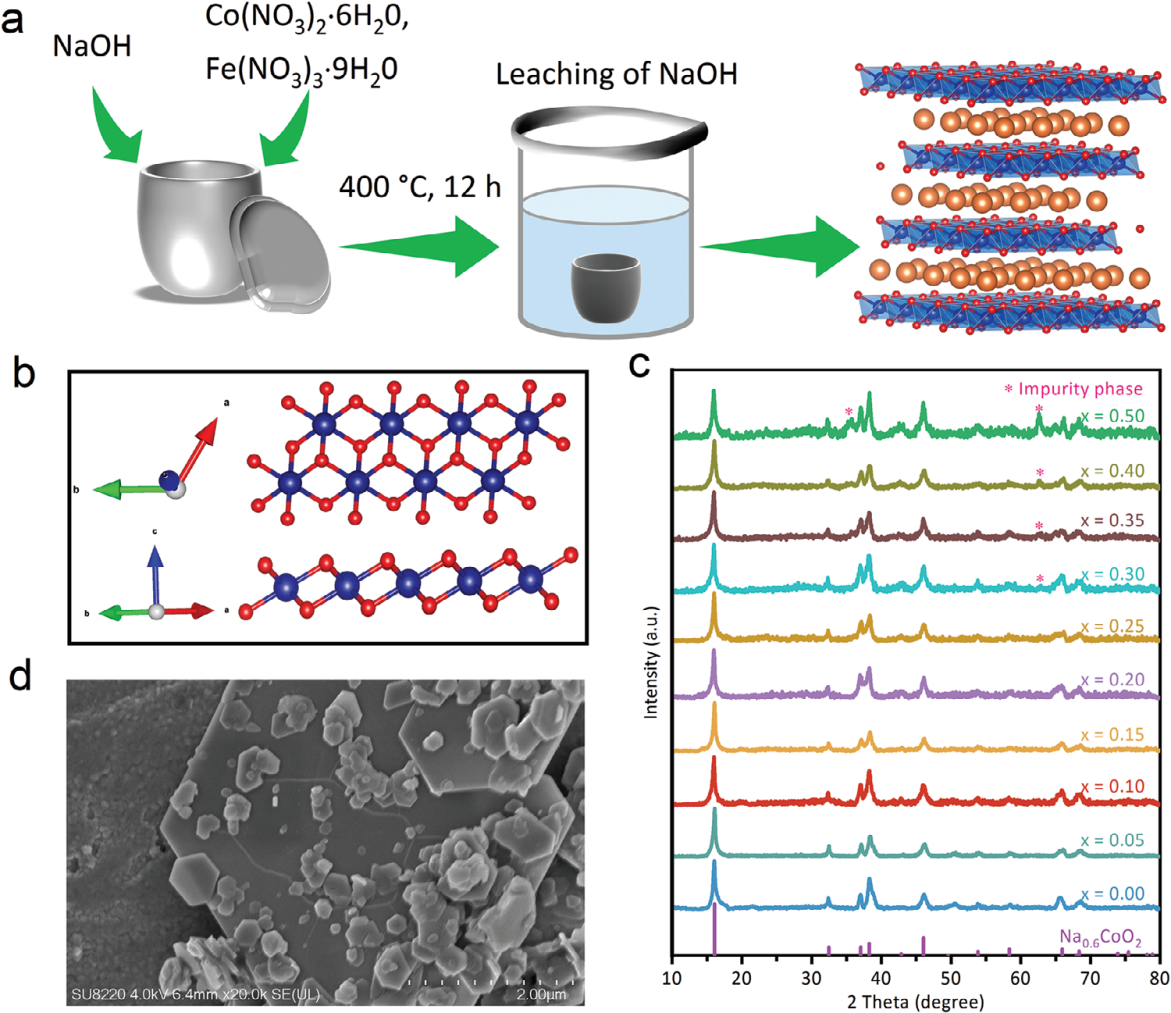
a) Schematic diagram of the synthetic method. b) XRD patterns of the as‐prepared samples. c) SEM image of NCF0.1. d) HRTEM image of NCF0.1.

The synthetic process is presented schematically in **Figure** **1**a. The di‐μ‐oxo bridged Co_Oh_ motifs are shown in Figure 1b with a plane view and side view. The X‐ray diffraction (XRD) peaks of Na_0.6_Co_1‐x_Fe_x_O_2_ (x = 0≈0.35) depicted in Figure 1c match well with the standard peaks in the previous report.^[28]^ Note that the impurity phase peaks emerge when x is greater than 0.25. As illustrated in Figure 1d, the scanning electron microscopy (SEM) image of NCF0.1 visually demonstrates the layer‐structured morphology. The high‐resolution transmission electron microscopy (HRTEM) image of NCF0.1 is shown in Figure S1 (Supporting Information), and the layered structure is substantially confirmed by the (001) and (003) crystal planes. We conduct inductively coupled plasma optical emission spectrometry (ICP‐OES) measurements to figure out the transition metal contents in the as‐prepared Na_0.6_Co_1‐x_Fe_x_O_2_. The results in Table S1 (Supporting Information) manifest that the molar ratio of Fe to Co is identical to the nominal stoichiometry. The energy‐dispersive X‐ray spectroscopy (EDS) elemental mappings of Na_0.6_CoO_2_ (denoted as NC) and NCF0.1, shown in Figure S2 (Supporting Information), further confirm the presence and homogeneous distribution of the noticed elements. These results strongly demonstrate the successful preparation of Na_0.6_Co_1‐x_Fe_x_O_2_.

**Figure 2:**
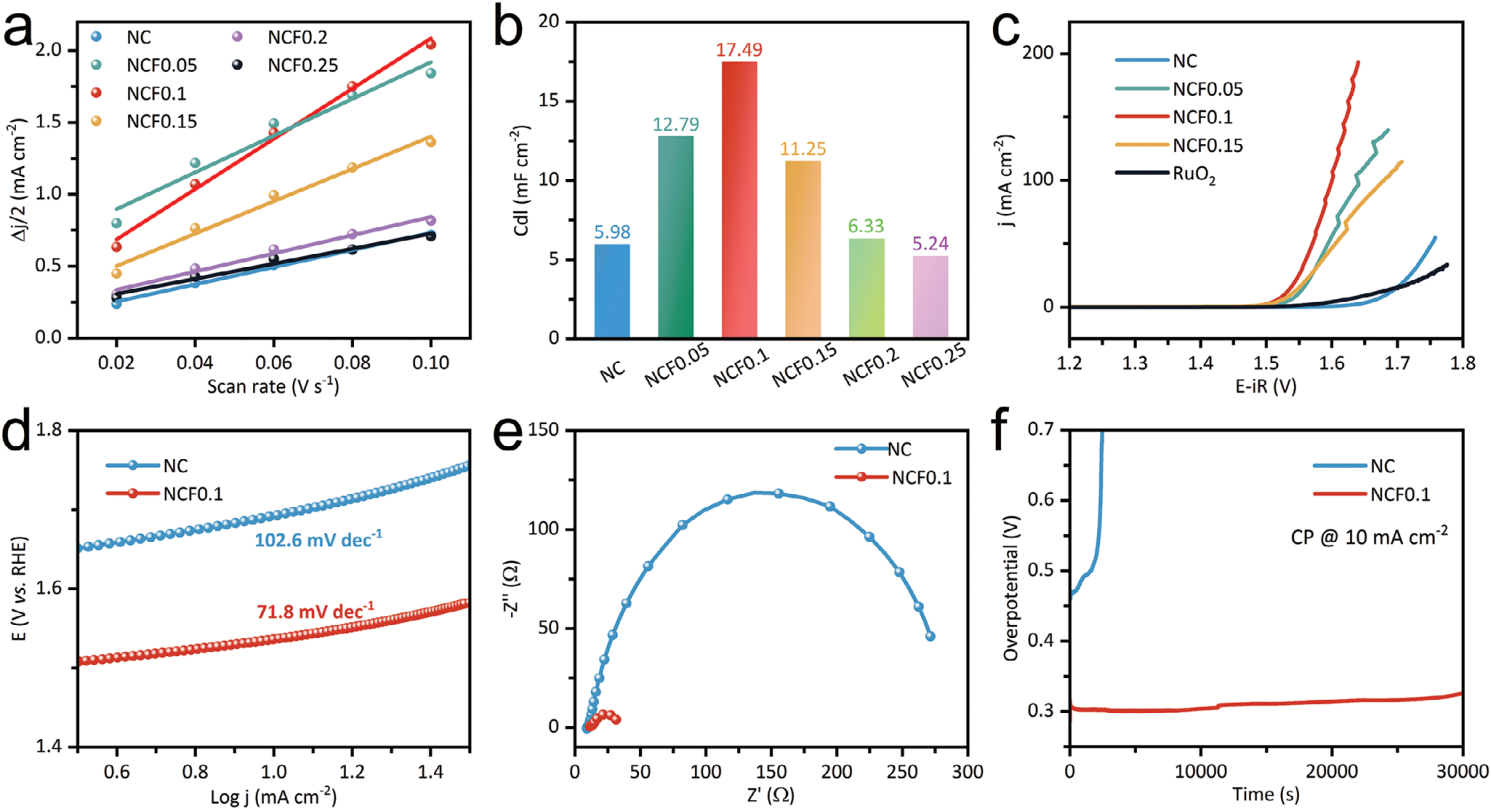
a) The linear fittings of the capacitive currents versus CV scans. b) The derived electrochemical double layer capacitances (Cdl). c) LSV curves. d) Tafel slopes (mV dec^−1^). e) Nyquist plots. f) The CP curves @ 10 mA cm^−2^.

Electrochemical tests were conducted for the NCFx under alkaline conditions (for details see Methods). The linear fittings of the capacitive currents versus cyclic voltammetry (CV) scan rates are plotted in **Figure** **2**a and the derived electrochemical double‐layer capacitances (Cdl) are shown in Figure 2b. Typically, the Cdl of NCF0.1 (17.49 mF cm^−2^) is much higher than that of pristine NC (5.98 mF cm^−2^), but the Cdl of the NCFx dropped dramatically with the increased Fe content for x > 0.1. Note that Cdl is the common measurement of the electrochemical surface area (ECSA), which is indicative of the fact that with the introduction of optimal amounts of Fe, NCF0.1 are abundantly enriched with electrochemically accessible active site. We infer that Fe activates inert Co sites on the basal plane, but does not act as active sites, this is responsible for the fact that ECSA continuously decreased with the increased Fe content for samples with x > 0.1. As seen in the linear sweep voltammetry (LSV) curves given in Figure 2c, NCF0.1 performs best among these oxides. In terms of the overpotential to afford a specific current density of 10 mA cm^−2^ (denoted as *η*_10_), NCF0.1 needs only 298 mV, which is much lower than NC (454 mV), commercial RuO_2_ (426 mV), and other reported Fe‐doped Na*_x_* CoO_2_ counterparts.^[^ ^25^, ^26^, ^27^ ^]^ The attenuation of OER activity of catalysts with high Fe content (0.1 ≤ x ≤ 0.25, Figure S3, Supporting Information) further excludes the possibility of Fe as active sites. The determination of the active sites in this case is consistent with some previous reports of Fe‐doped Co‐based oxides.^[^ ^29^, ^30^ ^]^ Reaction kinetics and charge‐transfer coefficient can be derived from the Tafel slope (Figure 2d) with 2.303RT/(0.5+n)F, where *n* is the number of electron‐transfer steps before the rate‐determining step (RDS), *F* is the Faraday constant, *R* is the ideal gas constant, and *T* is the thermodynamic temperature.^[31]^ Pristine NC catalyst delivers a Tafel slope of 102.6 mV dec^−1^, which suggests that RDS is the first electron‐transfer step. The Tafel slope decreases to 71.8 mV dec^−1^ for NCF0.1, indicating a mixed RDS involving the first and second steps,^[32]^ which further implies the activation of different reaction sites in NCF0.1. Moreover, the electrochemical impedance spectroscopic (EIS) test was conducted to understand the effect of the introduction of Fe on the charge transfer property of the catalysts. The derived Nyquist plot (Figure 2e) demonstrates the significantly reduced solid–liquid interfacial charge transfer resistance (*R*_ct_) for NCF0.1 compared with the bare NC electrocatalysts (circa 300 Ω), the accelerated interfacial reaction means the activated reaction sites in NCF0.1. The chronopotentiometry (CP) curves in Figure 2f indicate that the durability of NC drastically declined with time, while NCF0.1 demonstrates improved OER stability. Furthermore, the OER‐conditioned catalysts have conducted the CP test @ 20 mA cm^−2^ for 12 h, which are labeled as NC‐12 h and NCF0.1‐12 h. Figures S4 and S5 (Supporting Information) show the OER‐conditioned SEM and HRTEM images, suggesting the well‐preserved structural integrity and negligible surface reconstruction after OER. The OER‐conditioned electronic structure will be discussed in the following XPS analysis.

**Figure 3:**
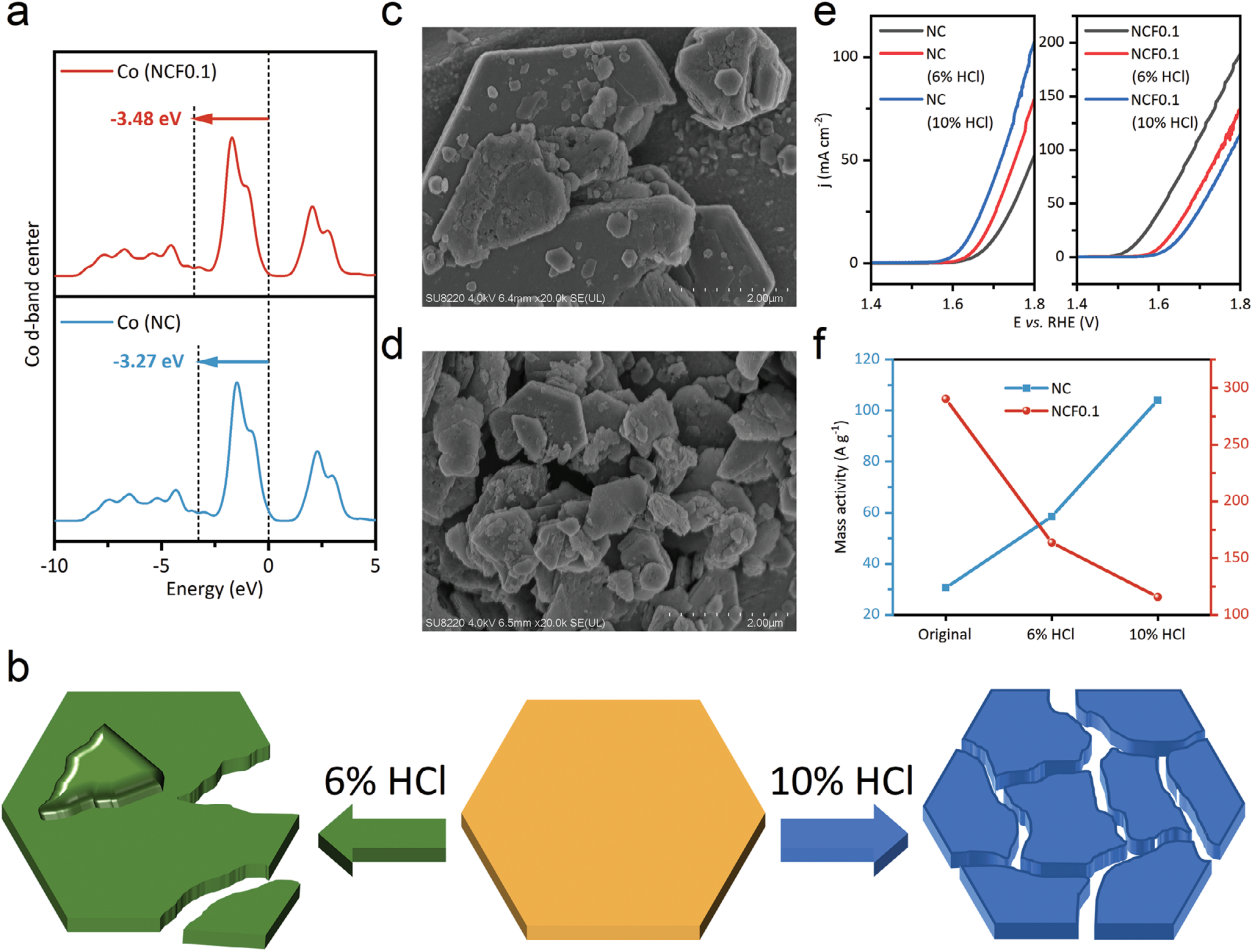
a) The Co d‐band center derived from the density of states (DOS) calculations. b) Schematic diagram of the etching process. SEM images of NCF0.1 with c) 6% HCl and d) 10% HCl etched. e) LSV curves of the pristine and the acid‐etched catalysts. f) The mass activity of the pristine and the acid‐etched catalysts.

As described in the above ECSA and Tafel slope analysis, the activity enhancement of Fe‐doped NC catalysts is attributed to the activation of metal sites in addition to the narrowed bandgap. Therefore, the electronic density of state (DOS) was calculated to determine the changes in the electronic structure of the catalysts following Fe substitution. The projected density of state (PDOS) of NCFx (x = 0, and 0.1) and the derived d‐band center energies are presented in **Figure** **3**a. The d‐band center energy is −3.27 eV for NC while it is −3.48 eV for NCF0.1. The Fe substitution in NC downshift the Co d‐band center far away from the Fermi level, which suggests the descended antibonding state energy of NCF0.1. Some researchers have suggested that the drop in the antibonding state will cause a decline in the energy of metal─O (M─O), which means weakened adsorption of oxygen intermediates.^[^ ^33^, ^34^ ^]^ Considering the inert nature of NC is the strong adsorption of the reaction intermediates on the large basal planes, we infer that Fe substitution in NC can optimize the adsorption of the intermediates on the basal planes, that is, activate the basal planes.

To verify this hypothesis, the catalysts were treated with acid‐etching and the change in OER activity was subsequently investigated. The acid‐etching will split the nanodisks into smaller pieces, which is schematically depicted in Figure 3b. SEM images (Figure 3c,d; Figure S6, Supporting Information) showed that the NCFx nanodisks partially crumbled after being immersed in 6% HCl for 9 h, and smaller fragments could be observed if a higher concentration of etching fluid (10% HCl) was applied. Note that the fragmented samples are stacked randomly, which makes it inevitable to cover part of the basal planes while the edge areas increase. Thus we can reasonably infer that as the concentration of etching fluid increases, the proportion of edge sites in the exposed surfaces increases.^[35]^ The O_2_‐temperature programmed desorption (O_2_‐TPD) results further corroborate the increase in exposed edge areas in 10% HCl‐treated samples (Discussed in Figure S7, Supporting Information). As expected, the OER activity of the acid‐etched NC was improved, owing to the increase in active edge sites. Interestingly, the *η*_10_ value has increased by ≈68 and ≈94 mV for the NCF0.1 etched with 6% and 10% HCl, respectively (Figure 3e). The derived mass activity of the catalysts before and after etching is shown in Figure 3f. The OER activity of NCF0.1 decreases obviously with the increase of the proportion of edge areas in the exposed surfaces, which confirms that the active surfaces of NCF0.1 for OER are basal planes rather than other edge areas. The Cdl values of the acid‐etched catalysts are depicted in Figure S8 (Supporting Information). As the introduction of acid leads to an increase in the exposed edge areas, the ECSA increases in NC, while drops in NCF0.1, which further validates that the active areas in NC are edge areas, but those in NCF0.1 are the basal planes.

**Figure 4:**
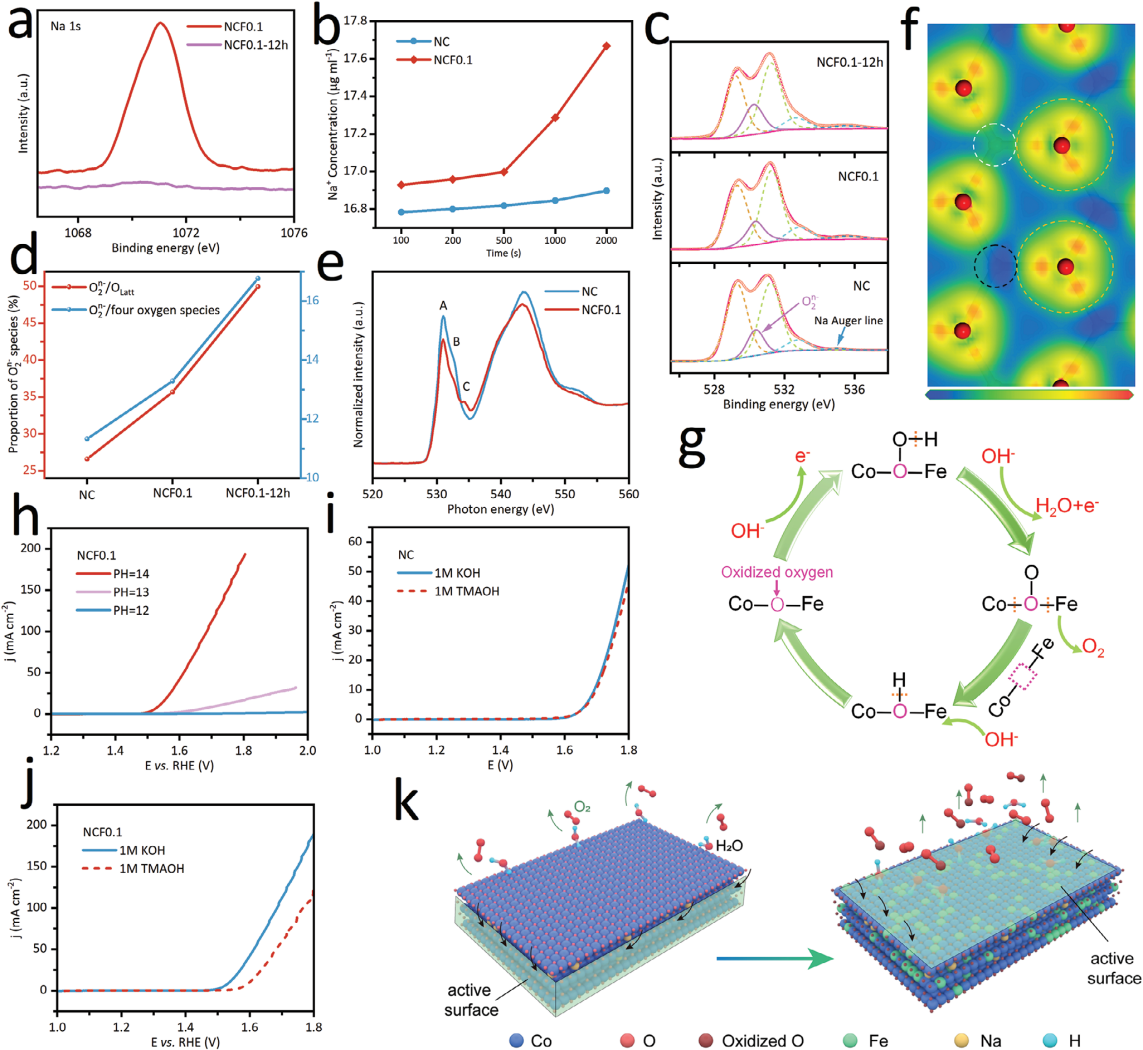
a) Na 1s XPS spectra. b) The Na^+^ concentration in the electrolyte measured by ICP‐OES. c) O 1s XPS spectra. d) The deconvoluted fraction of highly oxidative oxygen species (O_2_^n−^). e) O K‐edge XANES spectra. f) The calculated 2D charge density difference diagram. The atoms in white, black, and orange dotted circles are Fe, Co, and O, respectively. Red symbolizes the electron accumulation degree, and blue represents the electron depletion degree. g) Reaction steps and intermediates of the oxidized oxygen mechanism. h) LSV curves of NCF0.1 collecting in different pH‐valued KOH solutions. i) LSV curves of i) NC and j) NCF0.1 in 1 m KOH and 1 m TMAOH. k) The schematic diagram of modification.

The surface electronic states in the as‐prepared NCFx (x = 0≈0.15) are obtained from XPS and soft XAS measurements (as shown in Figures S9 and S10, Supporting Information), which discloses that there is nearly no change in the surface chemical state of Co and Fe in NCFx. By comparing with the XAS spectra of reference, the valence state of Co is higher than +3, while that of the substituted Fe is ≈+3. Thus, the unbalanced charge should be compensated by the O element in the system, based on the principle of electrical neutrality. The XPS spectra of OER‐conditioned samples in Figure S11 (Supporting Information) show that the electronic structures of the octahedral metals remain, while the Na 1s signal is almost undetectable after OER (**Figure** **4**a), which hints at the loss of Na. The ICP‐OES results in Figure 4b further confirm the extraction of Na in the OER process, and the extraction rate is significantly accelerated after the introduction of Fe. The rapid extraction of Na from NCF0.1 during OER will lead to further oxidation of oxygen for charge compensation. The oxygen species are first identified by the XPS O 1s spectra (Figure 4c), which can be deconvoluted into lattice oxygen species, highly oxidative oxygen species (O_2_^n−^), hydroxyl groups (OH^−^), and adsorbed molecular water (H_2_O) by the binding energy level.^[^ ^25^, ^36^ ^]^ The deconvolution results in Figure 4d show that the proportion of O_2_^n−^ species rises after the incorporation of Fe and in the OER process. In the O K‐edge XANES (Figure 4e), the edge at ≈528–538 eV is dominated by the hybridization of Co/Fe 3d and O 2p states.^[^ ^37^, ^38^ ^]^ The peaks “A” and “B” are ascribed to the t_2g_ and e_g_ states in metal 3d orbital where electrons are excited from O 1s level, respectively. It is worth noting that an obvious peak “C” emerges at 534.2 eV, which suggests the increased oxidative oxygen species in NCF0.1, cause a higher binding energy of core electrons in oxidative oxygen. As discussed in Figure S12 (Supporting Information), Hirshfeld charge analysis suggests that the introduction of Fe leads to oxidized oxygen in the doped CoO_2_ layer and a higher degree of oxidation of oxygen atoms around Fe. In addition, the calculated bond length in Table S2 (Supporting Information) indicates that the bond lengths of Fe─O and Co─O in NCF0.1 are 1.89 and 1.93 Å, respectively, which are shorter than the Co─O bond in NC (2.04 Å). The shortened bond length (attributable to oxygen oxidation), means a more direct M─O interaction, thus, facilitating oxygen evolution. The 2D charge density difference diagram in Figure 4f provides a more intuitive picture of the electronic structure. The electron depletion degree (blue) of Fe (white dotted circle) is much weaker than Co (black dotted circle), indicating the lower valence state of Fe. The O (orange dotted circle) around the Fe demonstrates a weaker electron accumulation degree (red) than that around the Co, which further confirms the oxygen oxidation.^[39]^ Therefore, we conclude that the introduction of Fe results in a relatively higher oxidation state of O in NCF0.1, and accelerated oxygen oxidation during OER.

Our previous work confirms that the oxidative oxygen species can serve as active sites independently, and break the scaling relationship limit (SRL), which originated from the adsorption of multi‐intermediates on the solo site.^[^ ^22^, ^40^ ^]^ The possible OER mechanism based on the oxidized oxygen is shown in Figure 4g. As indicated by the LSV curves in Figure 4h, NCF0.1 exhibits an extremely strong pH‐dependent characteristic, which implies nonconcerted proton‐electron transfers in the OER mechanism.^[^ ^41^, ^42^ ^]^ This observation reflects the change in RDS, consistent with the small Tafel slope value (71.8 mV dec^−1^) in NCF0.1. The identification of negatively charged oxygenated species (O_2_^−^ and O_2_^2−^) can provide direct evidence for the OER mechanism associated with the mentioned nonconcerted proton‐electron transfer. The tetramethylammonium cations (TMA^+^) can be employed as chemical probes to track these negative oxygenated intermediates during the OER, due to their strong electrostatic interaction.^[^ ^43^, ^44^ ^]^ Figure 4i,j presents the LSV curves in 1 m KOH and 1 m TMAOH solution systems, compared with the reaction in 1 m KOH, a significantly reduced OER activity of NCF0.1 in 1 m TMAOH can be observed due to the strong binding between TMA^+^ and O_2_^n−^, while the change in NC is negligible. These results indirectly make the above‐mentioned OER mechanisms responsible for the Fe‐incorporated sodium cobalt oxides. The schematic diagram of modification is illustrated in Figure 4k. The incorporation of Fe can optimize the adsorption of intermediates on the basal planes, and create oxidized oxygen. The shifted active area and the oxidized oxygen can synergistically improve OER performance.

## 3 Conclusion

In summary, we succeeded in synthesizing Na_0.6_Co_1‐_ *_x_* Fe*_x_* O_2_ with high Fe content without a protective atmosphere, and NCF0.1 with optimal OER activity was selected to study the reaction mechanism in depth. The active surface and electronic structure of the catalysts were studied through a wealth of experimental characterizations and theoretical calculations. The results consistently demonstrate that the introduction of Fe not only leads to the oxidation of oxygen but also the optimized adsorption of reaction intermediates, resulting in the transformation of the active surfaces from the edge to the large basal planes. The enriched active areas and the independent involvement of oxidative oxygen synergistically facilitate the OER process. The principles and mechanisms revealed in this work provide new insights for the study of the catalytical mechanism of heteroatom‐modified cobalt‐based oxides.

## Conflict of Interest

The authors declare no conflict of interest.

## Author Contributions

L.Y. and F.Z. conceived the project. X.B fabricated the samples, conducted most of the experimental characterization, processed the experimental data, and wrote the manuscript. F.T. performed the XAS characterization. F.Z completed the theoretical calculations. H.Q., W.J., and C.Z. revised the manuscript. All the authors discussed the results and contributed to the manuscript preparation.

## Supporting information

Supporting Information

## Data Availability

The data that support the findings of this study are available from the corresponding author upon reasonable request.
