# Supplementary material for: Activating the Basal Planes and Oxidized Oxygens in Layer‐Structured Na0.6CoO2 for Boosted OER Activity
Source: Adv Sci (Weinh). 2023 Nov 30;11(4):2305959. doi: 10.1002/advs.202305959 (PMC10811465; doi:10.1002/advs.202305959)
Supplement: Supplementary file 1 — Supporting Information [file ADVS-11-2305959-s001.pdf]

ADVANCED SCIENCE
Open Access

Supporting Information

for *Adv. Sci.*, DOI 10.1002/advs.202305959

Activating the Basal Planes and Oxidized Oxygens in Layer-Structured $Na_{0.6}CoO_2$ for Boosted OER Activity

Bing Xiong, Tingxin Fu, Qiuping Huang, Jianlin Wang, Zhangzhang Cui, Zhengping Fu and Yalin Lu*

Supporting Information

Activating the basal planes and oxidized oxygens in layer-structured $Na_{0.6}CoO_2$ for boosted OER activity

Bing Xiong [1], Tingxin Fu [1], Qiuping Huang[2 3], Jianlin Wang[2 3], Zhangzhang Cui[2 3], Zhengping Fu [1 2 3 *], Yalin Lu [1 2 3 *]

[1] CAS Key Laboratory of Materials for Energy Conversion, Department of Materials Science and Engineering, University of Science and Technology of China, Hefei 230026, China

[2] Anhui Laboratory of Advanced Photon Science and Technology, University of Science and Technology of China, Hefei 230026, China

[3] Synergetic Innovation Center of Quantum Information & Quantum Physics, Hefei National Research Center for Physical Sciences at the Microscale, University of Science and Technology of China, Hefei 230026, China

EXPERIMENTAL SECTION

Materials synthesis

All chemical reagents used in this work were analytical grade without further purification.

$Na_{0.6}Co_{1-x}Fe_xO_2$ (x=0, 0.05, 0.1, 0.15, 0.2, 0.25, 0.3, 0.35) powders were prepared by a one-pot synthesis method using NaOH as an alkaline solvent. First, 5 mM metal salt mixture ($Co(NO_3)_2 \cdot 6H_2O$ and $Fe(NO_3)_3 \cdot 9H_2O$ in specific molar ratio) and 14.5 g NaOH were added into a nickel crucible and reacted at 400 °C for 12 h. Then, the product with nickel crucible was soaked in deionized water for 40 h to leach NaOH. The resulting product was centrifuged with water and ethanol several times and finally dried at 70 °C for 10 h to obtain $Na_{0.6}Co_{1-x}Fe_xO_2$ oxides (named NC and NCFx).

Characterization

X-ray diffraction (XRD) curves were acquired with a conventional four-circle diffractometer (Rigaku, SmartLab, Cu K_α radiation). ESCALAB 250 X-ray photoelectron spectrometer with Al-K_α irradiation was conducted to perform X-ray photoelectron spectroscopy (XPS) profiles. Scanning electron microscopy (SEM, Hitachi-SU8220), transmission electron microscopy (TEM), and high-resolution transmission electron microscopy (HRTEM, JEM-2100F, 200KV) were performed to characterize the morphology of the obtained product. An inductively coupled plasma optical emission spectrometer (ICP-OES) was carried out for the determination of elemental content. Soft X-ray absorption spectroscopy (XAS) was collected at the National Synchrotron Radiation Laboratory BL12B-a (NSRL, Hefei, P. R. China). Determination of the proportions of different surfaces by O_2-TPD measurements (Micromeritics, AUTOCHEM II 2920).

Electrochemical measurements

The catalyst ink was prepared as follows: 10 mg catalyst and 2 mg carbon black were dispersed in a mixed 1100 μL solvent containing deionized water, isopropanol, and Nafion solution (V_{DI}

$_{water}$ / $V_{isopropanol}$ / V_{Nafion} = 7.5/2.5/1), with a further ultrasonication for at least 1 h. After a homogenized dispersion of catalysts formed, 3 uL of the ink was taken and spread onto the surface of the glassy carbon electrode to yield a final mass loading of 0.386 mg cm^{-2}. The catalyst ink was dropped on a glassy carbon electrode with a diameter of 3 mm as the working electrode, and platinum wire and saturated Ag/AgCl electrode were used as the counter and reference electrodes, respectively. RuO_2 is purchased from Sigma-Aldrich, and the RuO_2 electrode preparation method is the same as that of NCFx. All the electrochemical tests were carried out by a typical three-electrode system on an electrochemical workstation (CHI 760E, Chenhua, Shanghai) in a 1.0 M KOH electrolyte solution. Linear sweep voltammetry (LSV) curves were collected at a scan rate of 5 mV s^{-1}. Electrochemical impedance spectroscopy (EIS) was performed at 1.6 V versus reversible hydrogen electrode (RHE) over frequencies from 0.1 Hz to 10^5 Hz. All the current was normalized to the glassy carbon electrode's geometric surface area. The potentials versus Ag/AgCl were converted to versus RHE according to the Nernst Equation (1):

$$E_{\mathrm{RHE}} = E_{\mathrm{Ag/AgCl}} + E_{0\mathrm{Ag/AgCl}} + 0.059 \times \mathrm{pH} \qquad (1)$$

The $E_{iR\ corrected}$ is obtained following the equation: $E_{iR\ corrected} = E_{RHE} - iR_s$ (where i is the current, and R_s is the uncompensated ohmic solution resistance resolved from Nyquist plots.)

Calculations

Spin-polarized Density functional theory calculations were performed with the Cambridge Serial Total Energy Package (CASTEP) under the contract number 240A1NH. Hubbard U corrections were applied to d-electrons with the U_{eff} of 2.50 eV for and 4.00 eV for Co. The generalized gradient approximation (GGA) of Perdew-Burke-Ernzerhof (PBE) functionals with On-the-fly generation ultrasoft pseudopotentials were employed, and the cut-off energy was set to 598.70 eV. The $O_{24}Na_{7.2}Co_{11}Fe$ (NCF) cell was obtained by enlarging the $O_6Na_{1.8}Co_3$ (NC) cell to a 2×2×1 supercell, followed by replacing a Co atom with a Fe atom. The Brillouin zone for NC cell was sampled using 5×5×1 grids of k-points in the Monkhorst–Pack scheme, while that for NCF was 3×3×2. The convergence criteria were set to 1×10^{-5} eV $atom^{-1}$ for the total energy, 3×10^{-2} eV $Å^{-1}$ for the max force, 5×10^{-2} GPa for the max stress, and 1×10^{-3} Å for the max displacement respectively, while that for the self-consistent field was 1×10^{-6} eV $atom^{-1}$.[1]

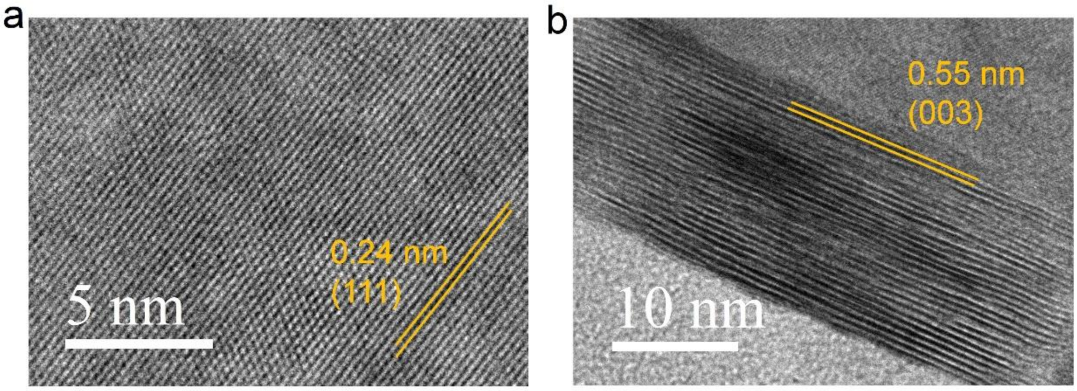

Figure S1. The high-resolution transmission electron microscopy (HRTEM) image of NCF0.1.

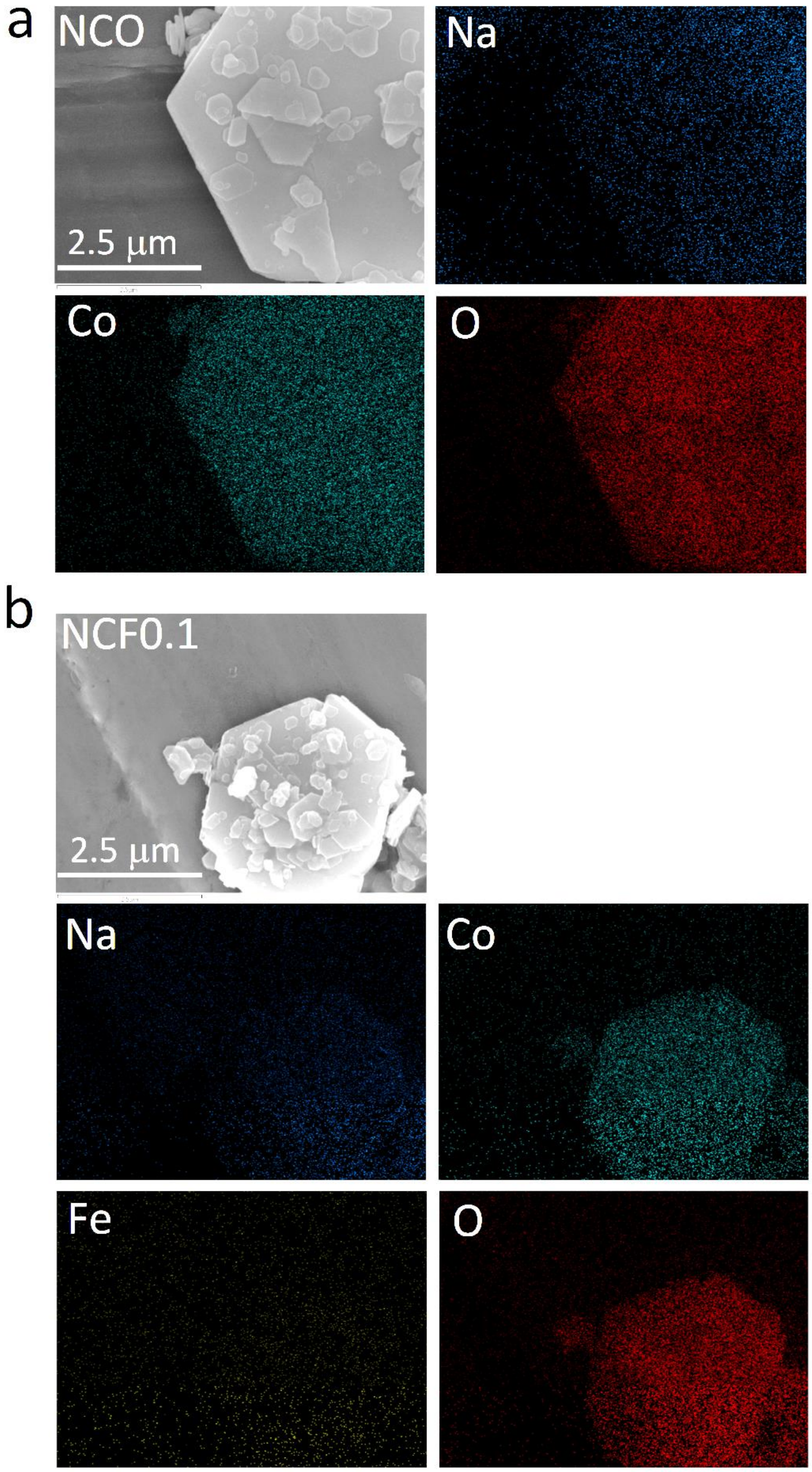

Figure S2. The energy-dispersive X-ray spectroscopy (EDS) elemental mappings of a) $Na_{0.6}CoO_2$ and b) $Na_{0.6}Co_{0.9}Fe_{0.1}O_2$.

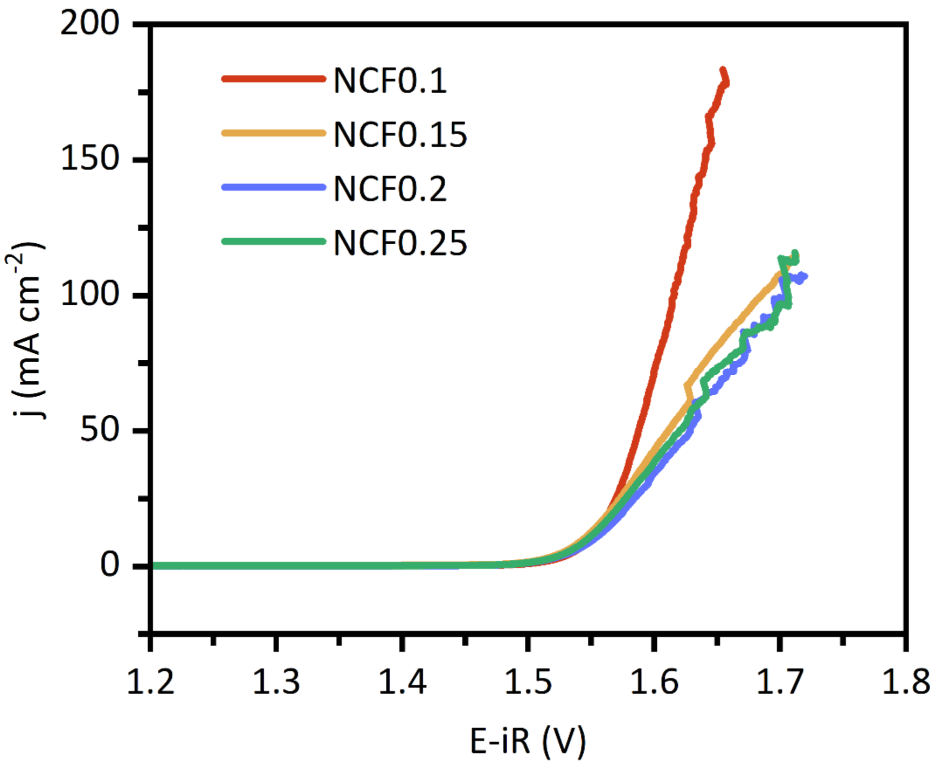

Figure S3. The LSV curves of $Na_{0.6}Co_{1-x}Fe_xO_2$($0.1 \leq x \leq 0.25$).

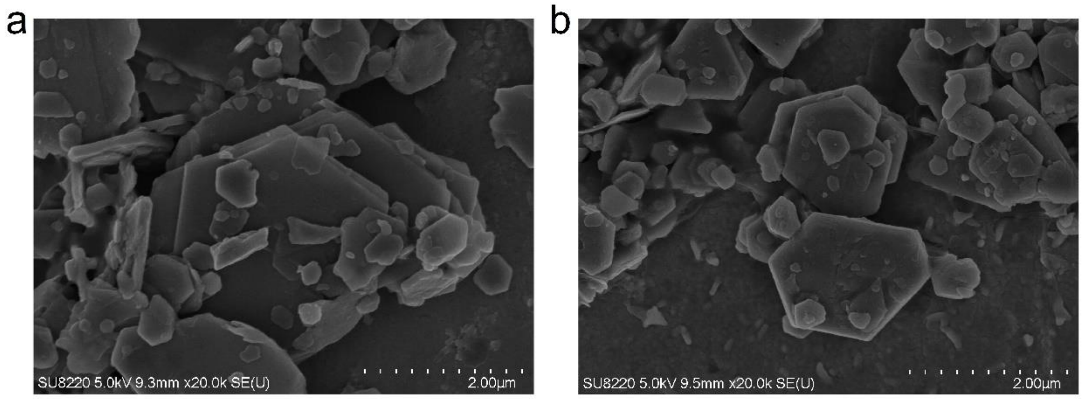

Figure S4. The OER-conditioned SEM images of (a) NC-12h and (b) NCF0.1-12h.

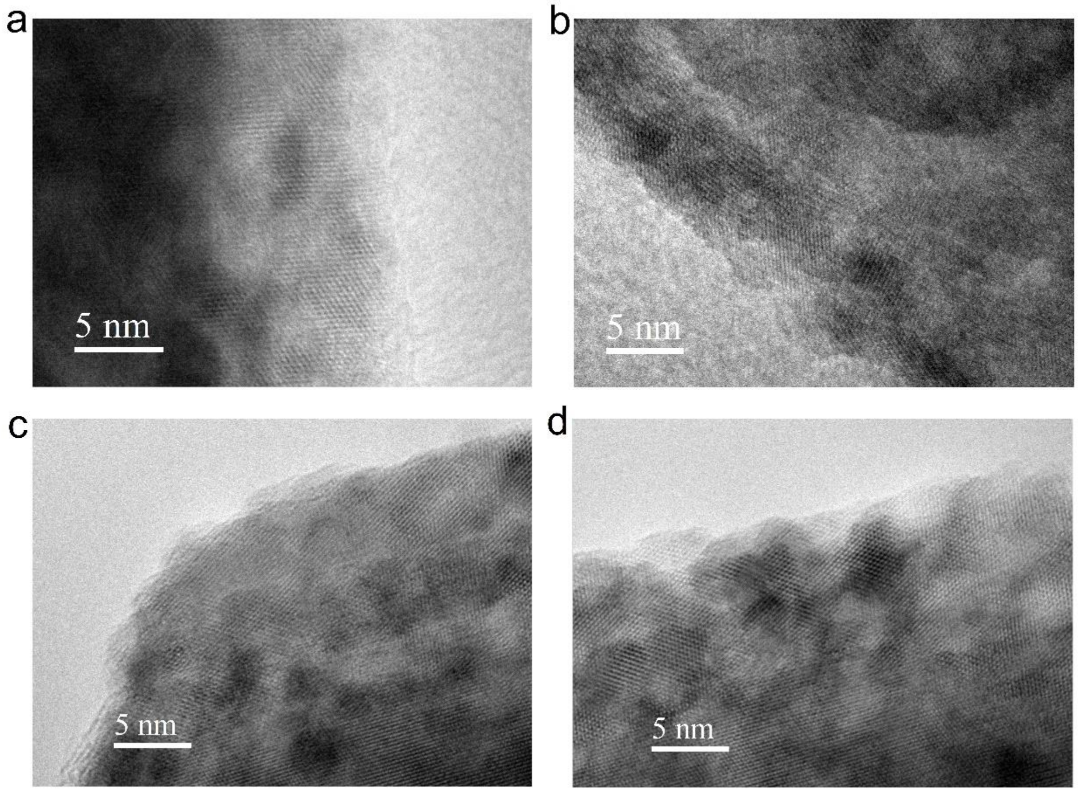

Figure S5. The OER-conditioned HRTEM images of (a-b) NC-12h and (c-d) NCF0.1-12h.

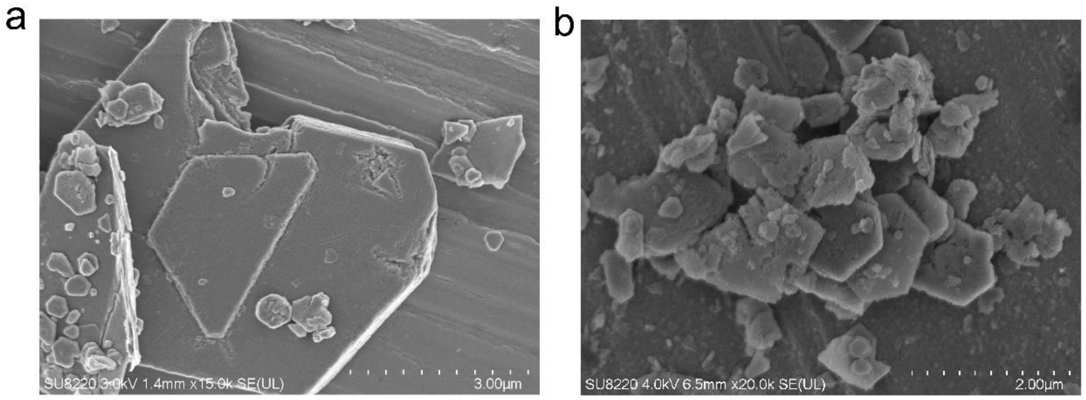

Figure S6. The SEM images of NC with (a) 6% HCl and (b) 10% HCl etched.

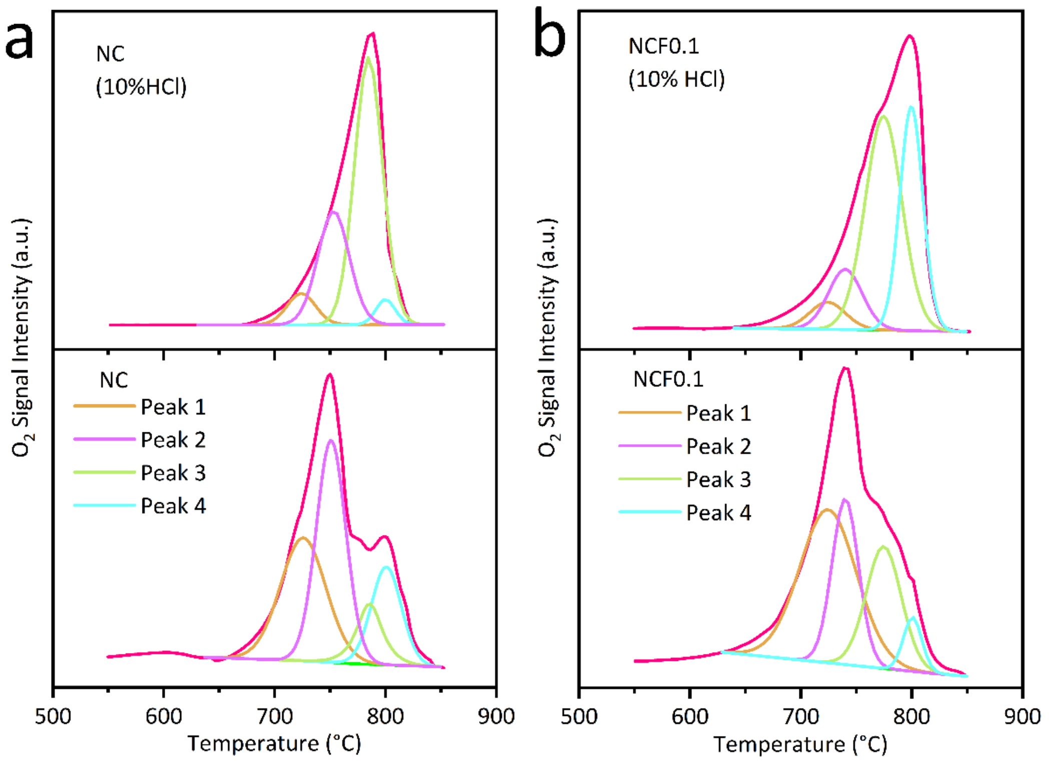

Figure S7. The O_2-temperature programmed desorption (O_2-TPD) profiles of (a) NC and NC (10%HCl), (b) NCF0.1 and NCF0.1 (10%HCl).

Discussion: The TPD measurements are widely used to recognize different surfaces of materials.[2-4] In general, the oxygen desorption peaks that appear below 500 °C can be ascribed to chemisorbed oxygen, while those occur after 600 °C are due to the desorption of lattice oxygen.[5-7] In this work, the TPD results are based on measurements in the high-temperature range, which corresponds to the desorption of lattice oxygen. As shown, each curve can be fitted to two regions, including the lower temperature region of Peaks 1 and 2 (named Region L), and the higher temperature region containing Peaks 3 and 4 (named Region H). Obviously, region L accounts for the main part in NC and NCF0.1, while Region H plays the dominant role in 10% HCl-treated samples. Sergio *et al.* proved by DFT calculations that compared with (012), (110), and (104) surfaces, (001) terminations demonstrate the lowest energy and are more inclined to lose oxygen in Li_xCoO_2.[8] Thus, we take Region L corresponding to the desorption of lattice oxygen with lower binding energy on the (001) basal planes, and Region H to the desorption of O on the edge areas. The fractions of edge areas significantly increase in the acid-treated samples, which is in line with our expectations.

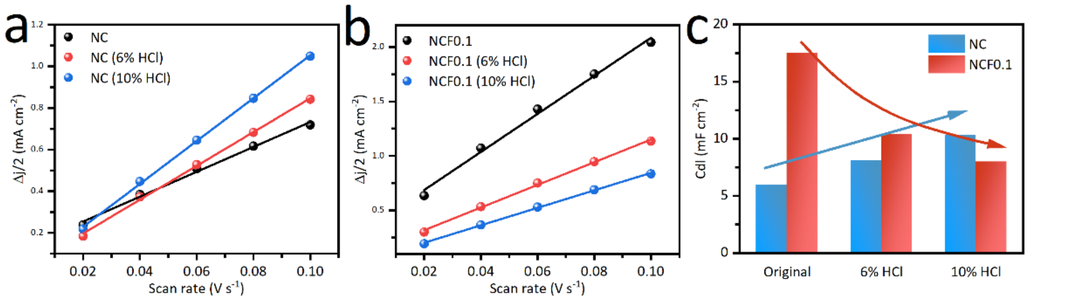

Figure S8. (a-b) The linear fittings of the capacitive currents versus CV scans. (c)The derived Cdl values of the catalysts before and after acid-etching.

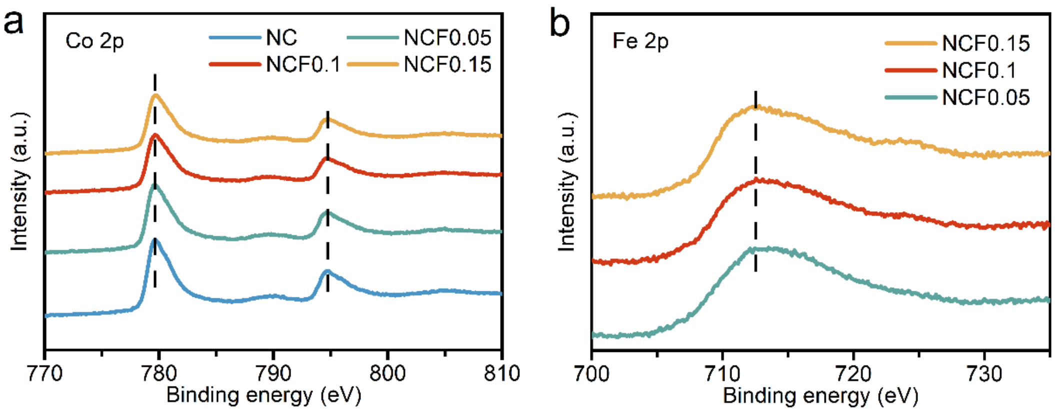

Figure S9. (a) Co 2p XPS spectra and (b) Fe 2p XPS spectra of the as-prepared catalysts.

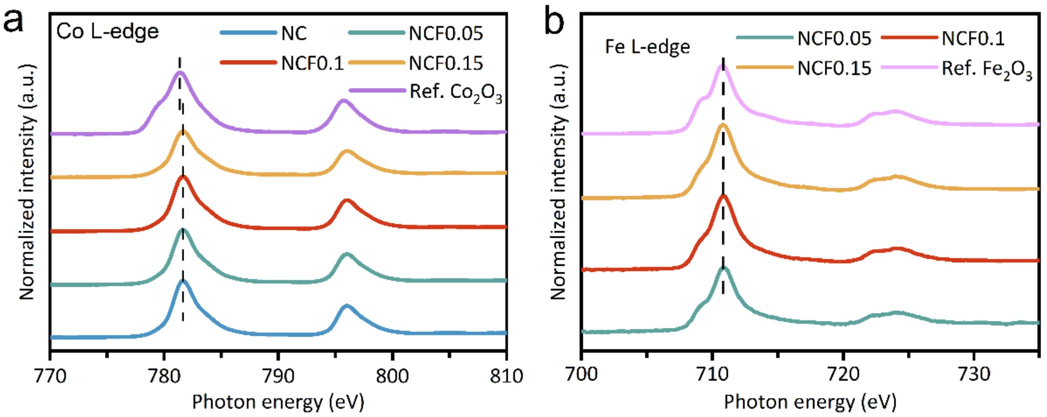

Figure S10. (a) Co L-edge and (b) Fe L-edge soft XAS spectra of the as-prepared catalysts.

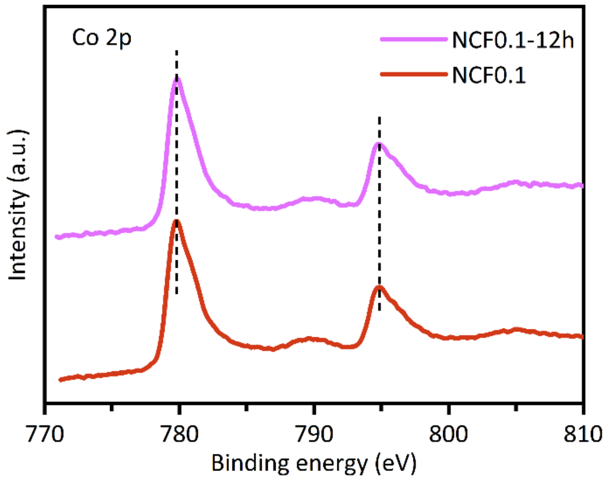

Figure S11. The Co 2p XPS spectra of NCF0.1 and NCF0.1-12h.

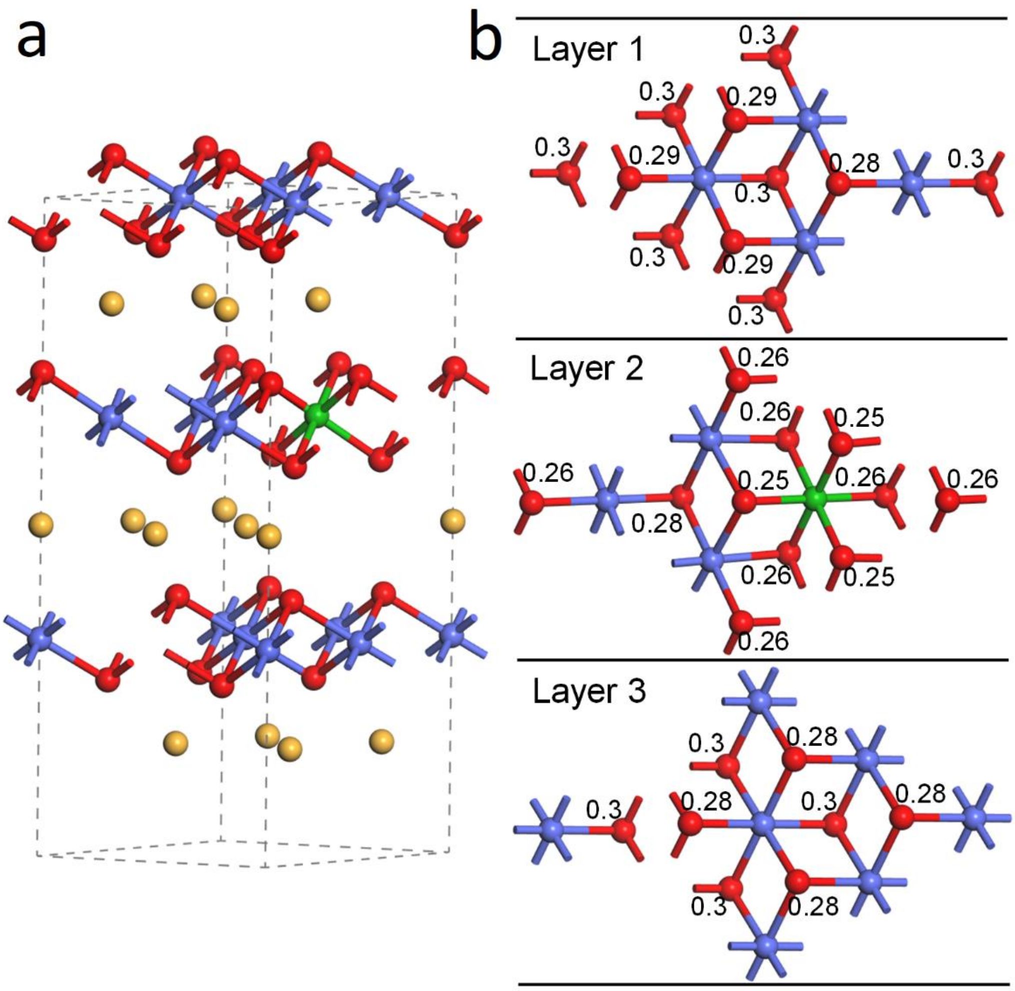

Figure S12. (a) The modeled structure of Fe-doped NC, the blue atoms represent Co, the red atoms represent O, the orange atoms represent Na, and the green atom represents Fe. (b) Hirshfeld charge analysis based on the structure in Figure a, the number near the oxygen atom represents the calculated charge accumulation of that oxygen atom.

Discussion: There are three CoO_2 layers in the structure, which are named Layer 1, Layer 2, and Layer 3 from top to bottom. The oxygen atoms in Layer 2 demonstrate a higher degree of oxidation compared to the other two CoO_2 layers. The O atoms around the Fe atom exhibit higher oxidation states with the exclusive consideration of Layer 2. Therefore, we can conclude that the introduction of Fe leads to oxidized oxygen in the doped CoO_2 layer and a higher degree of oxidation of oxygen atoms around Fe.

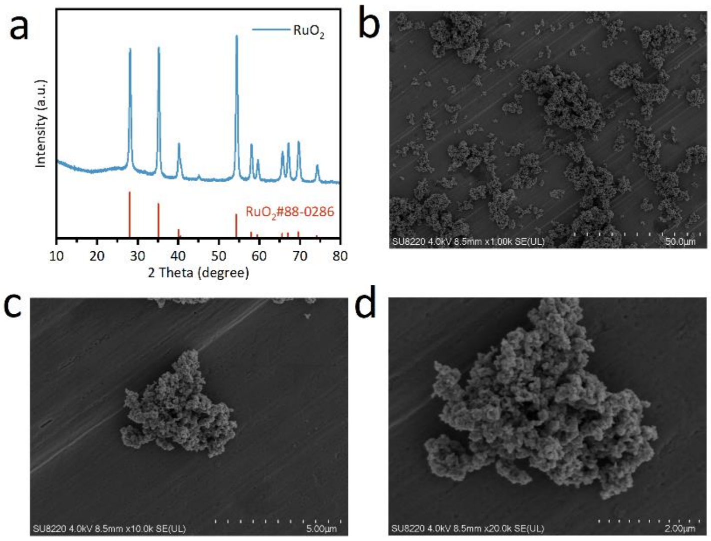

Figure S13. (a) XRD pattern and (b-d) SEM images of RuO_2.

Table S1. The inductively coupled plasma optical emission spectrometry (ICP-OES) measurements of the prepared $Na_{0.6}Co_{1-x}Fe_xO_2$.

Samples	Cobalt content (μg/ml)	Iron content (μg/ml)	Molar ratio of Fe/(Co+Fe)
NC	41.725	1.499	/
NCF0.05	31.608	1.499	4.8%
NCF0.1	30.158	3.054	9.7%
NCF0.15	25.785	4.136	14.5%

Table S2. The calculated metal-oxygen bond length.

Samples	Bond name	Bond length (Å)	Bond number
NC	O--Co	2.04266	3
NC	O--Co	2.04333	3
NCF0.1	O--Fe	1.89111	3
NCF0.1	O--Fe	1.89418	3
NCF0.1	O--Co	1.93043	3
NCF0.1	O--Co	1.93238	3
NCF0.1	O--Co	1.93407	3

NCF0.1	O--Co	1.93529	6
NCF0.1	O--Co	1.93657	3
NCF0.1	O--Co	1.93790	6
NCF0.1	O--Co	1.94003	3
NCF0.1	O--Co	1.94024	3
NCF0.1	O--Co	1.94309	6
NCF0.1	O--Co	1.94363	3
NCF0.1	O--Co	1.94403	3
NCF0.1	O--Co	1.94783	6
NCF0.1	O--Co	1.98770	3
NCF0.1	O--Co	1.99247	3
NCF0.1	O--Co	2.02275	6
NCF0.1	O--Co	2.03058	6

References

1. S. J. Clark, M. D. Segall, C. J. Pickard, P. J. Hasnip, M. I. J. Probert, K. Refson and M. C. Payne, *Zeitschrift für Kristallographie - Crystalline Materials*, 2005, **220**, 567-570.
2. F. J. Perez-Alonso, D. N. McCarthy, A. Nierhoff, P. Hernandez-Fernandez, C. Strebel, I. E. L. Stephens, J. H. Nielsen and I. Chorkendorff, *Angewandte Chemie*, 2012, **124**, 4719-4721.
3. M. J. van der Niet, A. den Dunnen, L. B. Juurlink and M. T. Koper, *J Chem Phys*, 2010, **132**, 174705.
4. M. J. van der Niet, A. den Dunnen, L. B. Juurlink and M. T. Koper, *Angew Chem Int Ed Engl*, 2010, **49**, 6572-6575.
5. W. Song, Z. Ren, S. Y. Chen, Y. Meng, S. Biswas, P. Nandi, H. A. Elsen, P. X. Gao and S. L. Suib, *ACS Appl Mater Interfaces*, 2016, **8**, 20802-20813.
6. X. Fu, Y. Huang, L. Yu, J. Yang, L. Li, Z. Jin, Y. Jin and S. Hu, *Environ Sci Pollut Res Int*, 2022, **29**, 44479-44489.
7. F. Cheng, J. Shen, B. Peng, Y. Pan, Z. Tao and J. Chen, *Nat Chem*, 2011, **3**, 79-84.
8. S. Posada-Pérez, G. Hautier and G.-M. Rignanese, *The Journal of Physical Chemistry C*, 2022, **126**, 110-119.
7. S. Posada-Pérez, G. Hautier and G.-M. Rignanese, *The Journal of Physical Chemistry C*, 2022, **126**, 110-119.
